# SFTA2 reduced colorectal cancer ferroptosis promoting metastasis through regulating EMT transition by degradation of Nrf2

**DOI:** 10.29219/fnr.v70.13267

**Published:** 2026-03-20

**Authors:** Jian Huang, Guihua Wei, Shengxun Mao

**Affiliations:** 1 Department of Gastrointestinal Surgery, The Second Affiliated Hospital, Jiangxi Medical College, Nanchang University, Nanchang, China; 2 Department of General Surgery, Jiujiang First People’s Hospital, Jiujiang, China; 3 Intensive Care Unit, Jiujiang First People’s Hospital, Jiujiang, China

**Keywords:** colon cancer, SFTA2, Nrf2, ferroptosis, mitochondrial damage

## Abstract

Colon cancer is a common malignancy of the digestive system, tumor disease, and its prevalence in China shows a consistently increasing trend. This study aimed to investigate the role of surfactant associated 2 (SFTA2) in colorectal cancer (CRC) and its molecular mechanism involving ferroptosis.

Colon cancer tissues were obtained from patients and normal volunteers from our hospital, and a mouse model of CRC was established using azoxymethane (AOM)/dextran sulfate sodium (DSS) induction.

SFTA2 expression was significantly up-regulated at both the messenger RNA (mRNA) and protein levels in CRC tissues and cell lines. Patients with high SFTA2 expression exhibited a shorter survival time compared to those with low SFTA2 expression. SFTA2 was found to be expressed in cancer cells of CRC patients, associated with key signaling molecules.

Sh-SFTA2 reduced cancer proliferation in the mice model of CRC. SFTA2 up-regulation promoted cell proliferation of CRC. SFTA2 down-regulation promoted cell proliferation of CRC. SFTA2 up-regulation reduced oxidative stress and ferroptosis of CRC. SFTA2 up-regulation reduced ferroptosis of CRC through mitochondrial damage-tricarboxylic acid cycle (TAC). SFTA2 down-regulation suppressed nuclear factor erythroid 2-related factor 2 (Nrf2) expression in the model of CRC. SFTA2 up-regulation reduced Nrf2 ubiquitination in the model of CRC. Nrf2 reversed the effects of si-SFTA2 on ferroptosis of CRC. Furthermore, SFTA2 down-regulation suppressed Nrf2 expression, while SFTA2 up-regulation decreased Nrf2 ubiquitination in the CRC model. Nrf2 was shown to reverse the pro-ferroptotic effects of si-SFTA2, indicating that SFTA2 activates the Nrf2 pathway by inhibiting its ubiquitination, thereby reducing mitochondrial damage and TCA cycle disruption in CRC.

SFTA2 induced the Nrf2 pathway to reduce mitochondrial damage-TAC of the CRC model through the inhibition of Nrf2 ubiquitination. SFTA2 is thus a potentiallyeffective therapeutic strategy for patients with CRC or other cancers.

## Popular scientific summary

- SFTA2 reduced colorectal cancer ferroptosis promoting metastasis by Nrf2.

Colorectal cancer (CRC) is one of the most prevalent malignancies worldwide, primarily affecting middle-aged and elderly individuals aged 30–69 years, with a higher incidence in males than females (1). Since 2000, CRC has shown a trend toward earlier onset, and its global incidence and mortality rates rank currently rank third and second, respectively (2). A 2015 global review highlighted significant geographical variation in both the incidence and mortality of CRC (3). In many regions, inadequate healthcare infrastructure contributes to low screening rates (4). The transition from chronic inflammation to cancer is considered to play an important role in CRC (5). Liver metastasis represents the most common cause of death among CRC patients (6). Current treatment strategies mainly involve surgery and chemotherapy (6). Although chemotherapy remains a cornerstone of management, most chemotherapeutic agents are associated with severe adverse effects, significant drug resistance, and high toxicity, which can lead to substantial collateral damage to patients (7).

Metastasis-associated in colon cancer-1 (MACC1) is a novel biomarker for colon cancer metastasis (8). Its significance stems from its role in regulating the hepatocyte growth factor (HGF)/c-Met signaling pathway. While this pathway is essential for normal physiological functions, including embryonic development and tissue repair, its dysregulation is a well-established driver of neoplastic progression, promoting the growth, proliferation, invasion, and metastasis of diverse cancer types (9, 10).

The c-Met roto-oncogene was first identified in 1984 by Cooper et al. as a transforming gene fragment cloned from N-methyl-N’-nitro-N-nitrosoguanidine (MNNG)-treated human osteosarcoma (MNNG-HOS) cells (11). Its encoded product is a transmembrane tyrosine protein kinase that serves as the sole high-affinity receptor for HGF. In normal physiology, c-Met is selectively expressed in specific epithelial tissues, including those of the liver, thyroid, digestive tract, as well as in fibroblasts and hematopoietic cells. Dysregulated c-Met expression is observed in a wide range of human malignancies, where it is closely associated with tumor initiation, progression, invasion, and metastasis (12).

The oncogenic functions of c-Met are primarily mediated through the following mechanisms: 1) Dysregulation of cell proliferation and apoptosis (13). Upon activation, the c-Met receptor phosphorylates and recruits phosphatidylinositol 3-kinase (PI3K). This catalyzes the production of phosphatidylinositol (3,4,5)-trisphosphate (PIP3), which in turn activates downstream effectors like protein kinase C (PKC) and initiates signaling cascades that ultimately promote cell proliferation and survival (14). 2) Promotion of tumor cell invasion. The HGF/c-Met axis facilitates invasion by acting on actin within the cytoskeleton to alter cell morphology and motility. It simultaneously enhances extracellular matrix degradation and weakens cell–cell adhesion, collectively increasing the migratory and invasive potential of tumor cells. 3) Induction of tumor angiogenesis. Angiogenesis is critical for both primary tumor growth and metastatic dissemination (15). Binding of HGF to c-Met on endothelial cells activates receptor tyrosine kinase, leading to phosphorylation of the β subunit. This triggers the PI3K and mitogen-activated protein kinase (MAPK) pathways, resulting in the upregulation of pro-angiogenic factors such as interleukin-8 (IL-8) and vascular endothelial growth factor (VEGF), thereby stimulating new blood vessel formation within the tumor (16, 17).

Ferroptosis is an iron-dependent form of regulated cell death characterized by the lethal accumulation of lipid peroxides and a breakdown of redox homeostasis (18). Its biochemical execution mechanism is distinct from other forms of cell death, such as apoptosis, necrosis, and autophagy (19, 20). As ferroptosis is often suppressed in cancers, its induction in tumor cells has emerged as a promising therapeutic strategy (21, 22). A central regulator of this process is glutathione peroxidase 4 (GPX4), which functions to detoxify iron-dependent lipid hydroperoxides (23). Consequently, inhibiting GPX4 results in unchecked lipid peroxidation, leading to ferroptotic cell death and the suppression of tumor cell proliferation (24).

Nuclear factor erythroid 2-related factor 2 (Nrf2) is a central transcriptional regulator critical for combating oxidative stress and maintaining cellular redox homeostasis. One of its key downstream effectors, GPX4 is a phospholipid hydroperoxidase that protects cells from ferroptosis by directly reducing lipid peroxides.

Surfactant associated 2 (SFTA2) demonstrates low expression in gastric cancer tissues, where its levels are closely associated with tumor size, lymph node metastasis, and patient prognosis. Functionally, SFTA2 overexpression inhibits the proliferation, migration, and invasion of gastric cancer cells. Similarly, in lung squamous cell carcinoma, SFTA2 is downregulated and linked to poor prognosis; its upregulation promotes cancer cell apoptosis and increases sensitivity to cisplatin. Parallel findings in lung adenocarcinoma show that SFTA2 downregulation facilitates tumor cell migration and invasion. In contrast, analysis of The Cancer Genome Atlas (TCGA) database reveals that SFTA2 is highly expressed in hepatocellular carcinoma. Given this context-specific dysregulation, we hypothesize that SFTA2 may also play a role in CRC development. This study therefore aims to investigate the function of SFTA2 in colon cancer and its underlying molecular mechanisms, with a specific focus on ferroptosis.

## Materials and methods

### AOM+DSS mice model of colon cancer and patients with colon cancer

The animal studies were authorized by the Animal Ethic Review Committees of our hospital. All animal experiments were strictly implemented in compliance with the National Institutes of Health (NIH) Guide for the Care and Use of Laboratory Animals and housed at 22–23°C, 55–60% humidity, in a 12-h light/dark cycle. Azoxymethane (AOM) + Dextran sulfate sodium (DSS) mice model mice were injected with 10 mg/kg of AOM (A5486, Sigma-Aldrich LLC. Shanghai, China), were induced by giving 2.0% dextran sulfate sodium (DSS, MP) in the drinking water for 7 days, drank normal water for 2 weeks, and were injected for three cycles.

We collected gene expression omnibus (GEO) data (GSE253699) to analyze the expression of SFTA2 as literature (23, 25).

Patients with colon cancer (number = 24) and normal volunteers (number = 7) were obtained from our hospital. This study was approved by the Ethics Committee of our hospital. Serum or tissue samples were collected and immediately stored at 80°C. Total ribonucleic acids (RNAs) were isolated with a RNA isolator total RNA extraction reagent (Takara) and Complementary DNA (cDNA) was synthesized using PrimeScipt reverse transcription (RT) Master Mix (Takara). Quantitative polymerase chain reaction (qPCR) were performed with the ABI Prism 7500 sequence detection. Relative levels of the sample messenger RNA (mRNA) expression were calculated and expressed as 2ΔΔCt.

### Cell culture and Animal model

NCM460, human colon tumor-116 (HCT-116), DLD-1, SW480, SW620HCT-116SW620 were cultured in Roswell Park Memorial Institute (RPMI) 1640 (Gibco) supplemented with 10% fetal bovine serum (FBS) (Gibco). HCT-116 cells were transfected with negative or SFTA2 using Lipofectamine 3000 (Invitrogen, CA). SW620 cells were transfected with si-nc or si-SFTA2 using Lipofectamine 3000 (Invitrogen, CA). The animal studies were authorized by the Animal Ethic Review Committees of our hospital. All animal experiments were strictly implemented in compliance with the NIH Guide for the Care and Use of Laboratory Animals. SW620 cells were transfected with negative or sh-SFTA2 lentivirus using Lipofectamine 3000 (Invitrogen, CA). All mice were inoculated with SW620 cells (1 × 107 cells).

### ELISA and cell viability assay

4-hydroxynonenal (4-HNE) (H268-1-2), CAT (A007-1-1), malondialdehyde (MDA) (A003-1-2), Superoxide Dismutase (SOD) (A001-3-2), glutathione (GSH) (A006-2-1), glutathione peroxidase (GSH-PX) (A005-1-2) were performed as described in a previous study (26). Cell viability was determined using a CCK-8 assay (C0037, Beyotime) as described in a previous study (27). Absorbance was measured using the Microplate Reader (Bio Tek, Winooski). EdU kit (C0075S, Beyotime) or lactate dehydrogenase (LDH) activity (C0016, Beyotime), Caspase3 (G01513), Caspase7 (H080) and caspase9 (G01811) were quantified using a commercial reagent kit (Nanjing Jiancheng Bioengineering Research Institute) and Absorbance was measured at 450 nm using a fluorescent reader (Synergy H1 Microplate Reader, Bio Tek, Winooski).

### Western Blotting Analysis and Immunofluorescence

Western Blotting Analysis and Immunofluorescence were executed as stated in the literature (27). MFAP4 (ab169757, 1:1000, Abcam), FAK (ab40794, 1:1000, Abcam), GPX4 (ab125066, 1:1000, Abcam), β-actin (1:10000, AC028, Company ABclonal, Inc.) and Anti-Rabbit IgG (1:5000, GB23303, Servicebio) were used in this study. Protein was measured using an BeyoECL Plus kit (P0018S) and analyzed using an Image Lab 3.0 (BioRad Laboratories, Inc.). MFAP4 (ab169757, 1:1000, Abcam), and FAK (ab40794, 1:1000, Abcam) was used for immunofluorescence analyses.

### Histological, immunohistochemical, and immunofluorescence analyses and electron microscopy

For immunohistochemical and immunofluorescence analyses, mouse tissue samples were fixed in 4% paraformaldehyde and stained as described in previous studies (28). Samples were observed under a fluorescence microscope (Zeiss Axio Observer A1, Germany) and a transmission electron microscope (80 kV) (Hitachi H7650, Tokyo, Japan) as described in a previous study (27).

### Statistical analysis

*P* < 0.05 was considered significant and evaluated using Student’s *t*-test or one-way analysis of variance (ANOVA) followed by Tukey’s post-test. Data were expressed as mean ± standard deviation (SD).

## Results

### SFTA2 expression in CRC model

**Fig. 1:**
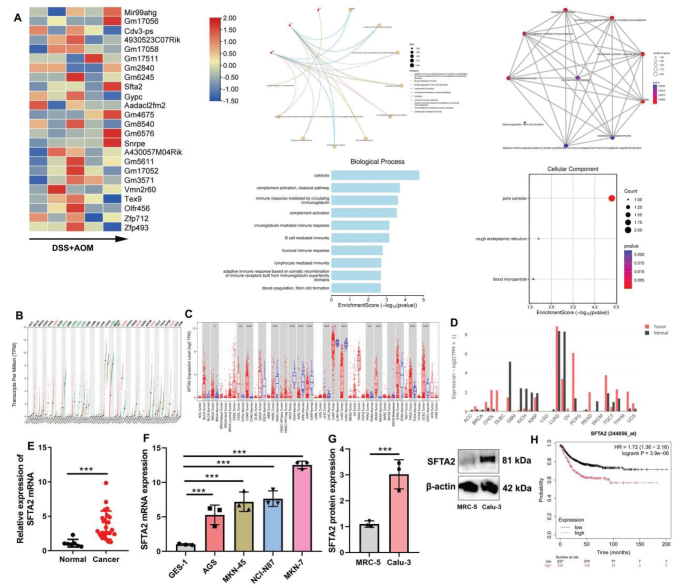
SFTA2 expression in the model of colorectal cancer.SFTA2 expression in DSS + AOM-induced colorectal cancer mice model using gene chip (A); SFTA2 mRNA expression in patients with cancer (B, C, D); SFTA2 mRNA expression in patients with colorectal cancer (E); SFTA2 mRNA and protein expressions in colorectal cancer cells lines (F, G); Survival time in patients with colorectal cancer (H). ***P < 0.001. Data were expressed as mean ± SD; the number of patients = 24; the number of normal group = 6; the number of vitro model = 3.

First, this study explored the disease targets for the occurrence and progression of CRC using gene chip (Fig. 1A). Analysis revealed SFTA2 expression across various human tumor samples (Fig. 1B). Subsequently, SFTA2 was found to be significantly up-regulated at both the mRNA and protein levels in clinical CRC tissues and established CRC cell lines, compared to corresponding normal controls (Fig. 1B–G). Concomitantly, survival analysis indicated that patients with high SFTA2 expression had a significantly shorter overall survival time than those with low SFTA2 expression (Fig. 1H). Therefore, SFTA2 may have a specific correlation with patient prognosis.

### SFTA2 expression CRC cells

**Fig. 2:**
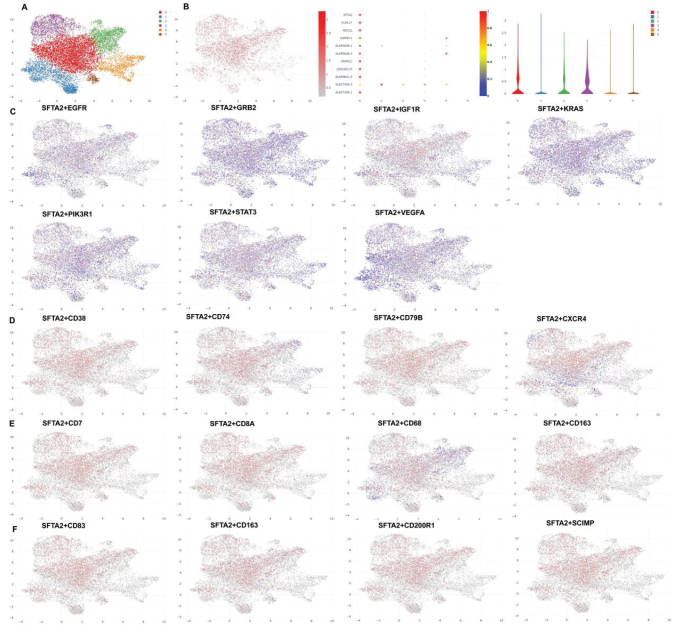
SFTA2 expression in colorectal cancer cells.Single-cell sequencing data for SFTA2 expression (A, B), SFTA2 expression in cancer cells (EGFR / GRB2 / IGF1R / KRAS / PIK3R1 / STAT3 / VEGFR, C); B cells (CD38/ CD74/ CD79B/ CXCR4, D), T cells (CD7/ CD8A/ CD68/ CD163, E), and macrophage (CD83/ CD163/ CD201R1/SCIMP, F) in colorectal cancer patients.

To elucidate the role of SFTA2 in CRC, we performed single-cell RNA sequencing analysis. Evaluation of SFTA2 expression across the tumor microenvironment revealed its specific presence in malignant epithelial cells (Fig. 2A–B). Further characterization confirmed that SFTA2 was predominantly expressed within a cancer cell subpopulation positive for established oncogenic markers (EGFR / GRB2 / IGF1R / KRAS / PIK3R1 / STAT3 / VEGFR) (Fig. 2C). In contrast, SFTA2 expression was undetectable in defined immune cell populations, including B cells (marked by CD38, CD74, CD79B, CXCR4), T cells (CD7, CD8A), and macrophages (CD68, CD163, CD83, CD201R1, SCIMP) (Fig. 2D–F).

### Sh-SFTA2 reduced cancer proliferation in mice model of CRC

**Fig. 3:**
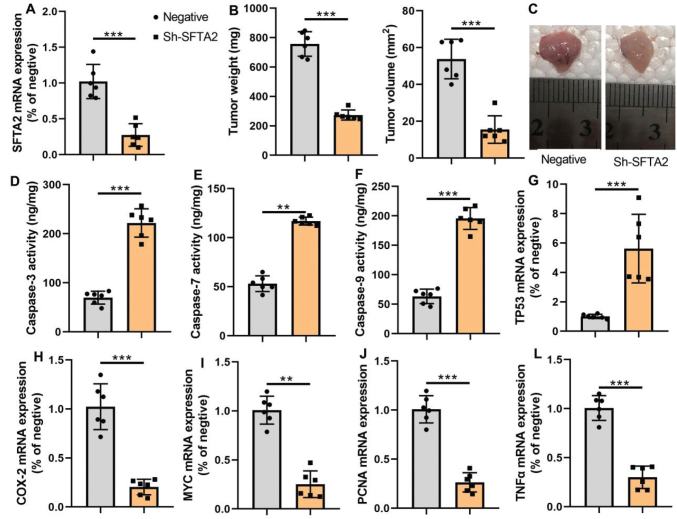
Sh-SFTA2 reduced cancer proliferation in mice model of colorectal cancer. SFTA2 expression (A), tumor weight/volume (B), cancer tissue (C), caspase-3/7/9 activity levels (D, E, F), TP53 mRNA expression (G), Cox2/TNF-α/MYC mRNA expression (H, I, J) in the mice model. **P < 0.01, ***P < 0.001. Data were expressed as mean ± SD; the number of mice model = 6.

To investigate the functional consequences of SFTA2 loss, we employed an shRNA-mediated knockdown approach in a murine CRC model (Fig. 3A). SFTA2 down-regulation markedly inhibited tumorigenesis, resulting in decreased tumor volume and weight. This anti-tumor effect was associated with a pro-apoptotic phenotype, demonstrated by elevated caspase-3/7/9 activity. A concerted alteration in key gene expression was also observed, characterized by reduced mRNA levels of the oncogenic and inflammatory markers Cox2, TNF-α, and Myc, and a concurrent increase in the tumor suppressor TP53 (Fig. 3B–L).

### SFTA2 up-regulation promoted cell proliferation of CRC

**Fig. 4:**
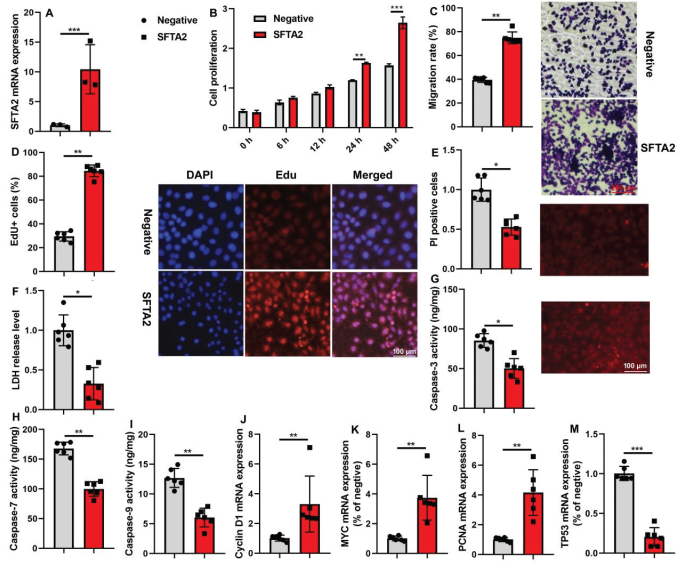
SFTA2 up-regulation promoted cell proliferation of colorectal cancer. SFTA2 expression (A), cell growth (B), Migration (C), Edu positivity (D), PI levels (E), LDH activity (F), caspase-3/7/9 activity levels (G, H, I), Cyclin D1/ myc /PCNA/ TP53 mRNA expression (J, K, L, M) in colorectal cancer cells. *P < 0.05, **P < 0.01, ***P < 0.001. Data were expressed as mean ± SD; the number of vitro model = 3 or 6.

Following the successful up-regulation of SFTA2 mRNA in CRC cells (Fig. 4A), functional assays confirmed its role in promoting cell growth (Fig. 4B). Concurrently, PRSS22 overexpression enhanced malignant phenotypes, as indicated by increased EdU incorporation and cell migration, alongside reduced LDH activity and PI staining (Fig. 4C–F). At the molecular level, the up-regulation of SFTA2 reduced caspase-3/7/9 activity levels, inhibited TP53 mRNA expression, and increased Cyclin D1/ myc /PCNA mRNA expression levels in CRC cells in (Fig. 4G–M).

### SFTA2 down-regulation promoted cell proliferation of CRC

**Fig. 5:**
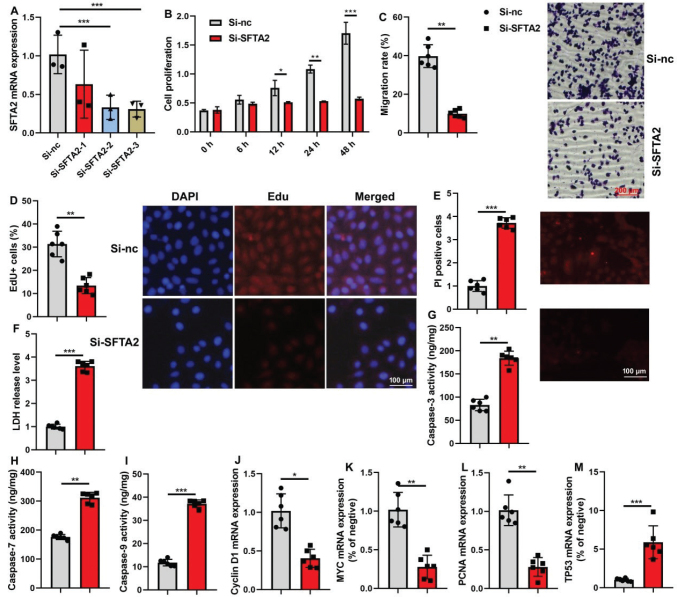
SFTA2 down-regulation promoted cell proliferation of colorectal cancer. SFTA2 expression (A), cell growth (B), Migration (C), Edu positivity (D), PI levels (E), LDH activity (F), caspase-3/7/9 activity levels (G, H, I), Cyclin D1/ myc /PCNA/ TP53 mRNA expression (J, K, L, M) in colorectal cancer cells. *P < 0.05, **P < 0.01, ***P < 0.001. Data were expressed as mean ± SD; the number of vitro model = 3 or 6.

Consistent with a tumor-promoting role, knockdown of SFTA2 decreased its mRNA expression and subsequently suppressed CRC cell growth (Fig. 5A–B). Phenotypically, SFTA2 down-regulation led to reduced Edu incorporation and cell migration, while conversely increasing LDH activity and PI staining, indicating enhanced cell damage and death (Fig. 5C–F). Concomitantly, at the molecular level, SFTA2 silencing promoted apoptosis by elevating caspase-3/7/9 activity, increased TP53, and down-regulated key proliferation markers (Cyclin D1, Myc, PCNA) (Fig. 5G–M).

### SFTA2 up-regulation reduced oxidative stress of CRC

**Fig. 6:**
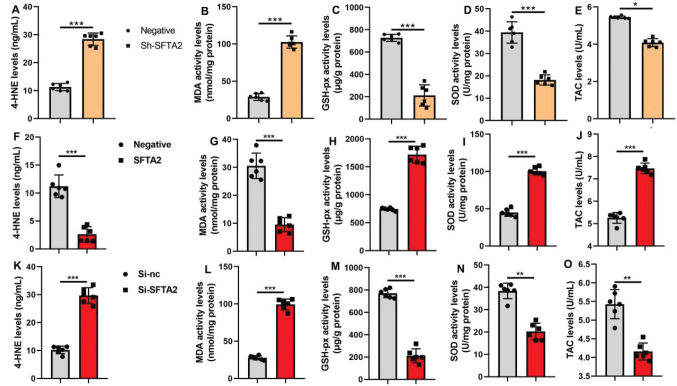
SFTA2 up-regulation reduced the oxidative stress of colorectal cancer. 4-HNE, MDA, SOD, GSH-PX, and TAC activity levels (A, B, C, D, E) in the tumor tissue of mice model; 4-HNE, MDA, SOD, GSH-PX, and TAC activity levels (F, G, H, I, J) in the vitro model by SFTA2 up-regulation; 4-HNE, MDA, SOD, GSH-PX, and TAC activity levels (K, L, M, N, O) in the vitro model by SFTA2 down-regulation. *P < 0.05, **P < 0.01, ***P < 0.001. Data were expressed as mean ± SD; the number of vitro model = 6.

In the murine CRC model, shRNA-mediated knockdown of SFTA2 significantly promoted oxidative stress, as indicated by elevated levels of 4-HNE and MDA, alongside reduced activity of the antioxidant enzymes SOD and GSH-Px, and a decrease in tricarboxylic acid cycle (TAC) (Fig. 6A–E). Conversely, in vitro experiments demonstrated that SFTA2 overexpression attenuated oxidative damage by suppressing 4-HNE and MDA levels, while enhancing SOD and GSH-Px activity and TAC (Fig. 6F–J). Consistently, SFTA2 knockdown in cultured cells recapitulated the in vivo findings, leading to increased 4-HNE/MDA and decreased antioxidant parameters (SOD, GSH-Px, TAC) (Fig. 6K–O).

### SFTA2 up-regulation reduced ferroptosis of CRC

**Fig. 7:**
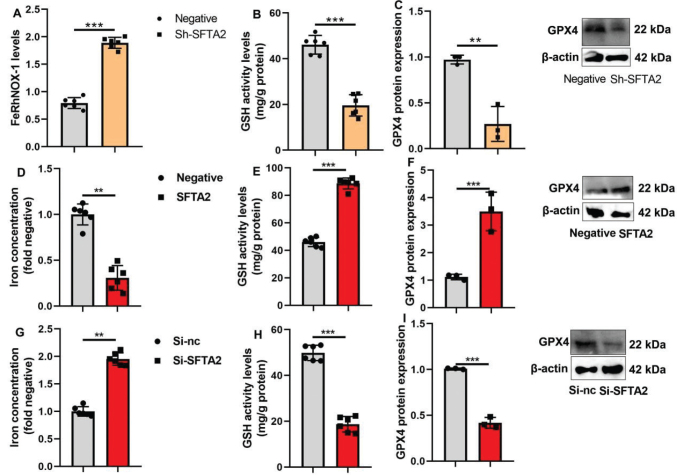
SFTA2 up-regulation reduced ferroptosis of colorectal cancer. FeRhNOX-1 levels, GSH activity level, and GPX4 protein expression (A, B, C) in tumor tissue of mice model; iron concentration level, GSH activity level, and GPX4 protein expression (D, E, F) in vitro model by SFTA2 up-regulation; iron concentration level, GSH activity level and GPX4 protein expression (G, H, I) in vitro model by SFTA2 down-regulation. *P < 0.05, **P < 0.01, ***P < 0.001. Data were expressed as mean ± SD; the number of mice model = 6; the number of vitro model = 3 or 6.

While SFTA2 has been implicated in CRC, its role in regulating mitochondrial oxidation-induced ferroptosis remains unclear. To investigate this, we first examined key ferroptosis markers in a murine model and found that SFTA2 knockdown increased levels of Fe² and HMOX1, while reducing GSH activity and GPX4 protein expression in tumor tissues (Fig. 7A–C). Conversely, in vitro experiments showed that SFTA2 overexpression decreased intracellular iron concentration and enhanced both GSH activity and GPX4 expression (Fig. 7D–F). . Supporting this, SFTA2 knockdown in cultured cells increased iron accumulation and suppressed the GSH/GPX4 axis (Fig. 7G–I), collectively indicating that SFTA2 inhibits ferroptosis by maintaining iron homeostasis and antioxidant capacity.

### SFTA2 up-regulation reduced ferroptosis of CRC through mitochondrial damage-TAC

**Fig. 8:**
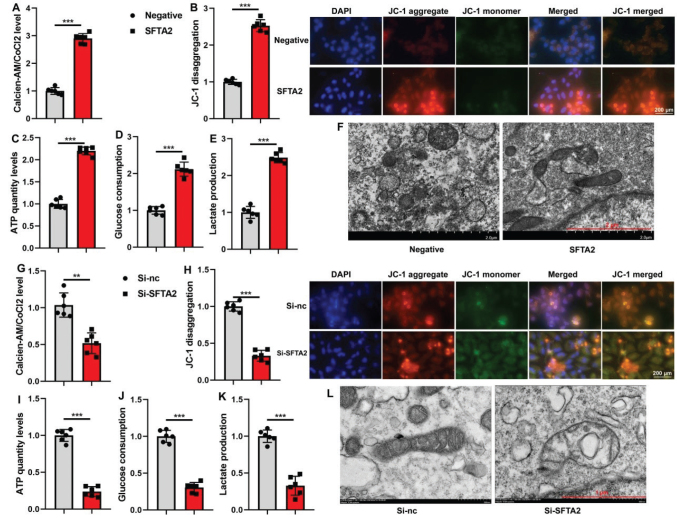
SFTA2 up-regulation reduced ferroptosis of colorectal cancer through the mitochondrial damage-tricarboxylic acid cycle (TAC). Mitochondria CoCl2 levels (A), JC-1 assay (B), ATP quantity levels (C), Glucose consumption (D), Lactate production (E), Mitochondrial damage (electron microscope, F) in vitro model by SFTA2 up-regulation; Mitochondria CoCl2 levels (G), JC-1 assay (H), ATP quantity levels (I), Glucose consumption (J), Lactate production (K), Mitochondrial damage (electron microscope, L) in vitro model by SFTA2 down-regulation. **P < 0.01, ***P < 0.001. Data were expressed as mean ± SD; the number of in vitro model = 3 or 6.

We next assessed the effect of SFTA2 on mitochondrial function. In CRC cells, SFTA2 up-regulation enhanced mitochondria membrane potential (as indicated by increased JC-1 aggregation and CoCl_2_ levels) and boosted TAC cycle activity, reflected by elevated ATP production, glucose consumption, and lactate output. This was accompanied by a significant reduction in mitochondrial damage (Fig. 8A–F). Conversely, SFTA2 knockdown impaired mitochondrial integrity, decreasing membrane potential and TCA cycle metrics, while exacerbating mitochondrial damage (Fig. 8G–L).

### SFTA2 down-regulation suppressed Nrf2 expression in the model of CRC

**Fig. 9:**
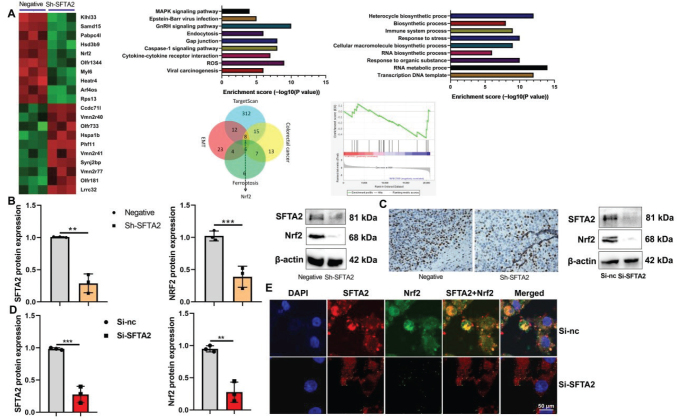
SFTA2 down-regulation suppressed Nrf2 expression in the model of colorectal cancer. Heat map (A), SFTA2/ Nrf2 protein expression (B), Nrf2 expression (Immunohistochemistry, (C) in mice mode; SFTA2/ Nrf2 protein expression (D), SFTA2/ Nrf2 protein expression (immunofluorescence, E) in vitro model by SFTA2 down-regulation. **P < 0.01, ***P < 0.001. Data were expressed as mean ± SD; the number of vitro model = 3.

To systematically investigate the role of SFTA2 in mitochondrial oxidation-induced ferroptosis, we first employed a gene chip screening approach (Fig. 9A). Results from a mouse model of CRC demonstrated that Sh-SFTA2 effectively suppressed the protein expression of both SFTA2 and Nrf2 in tumor tissue (Fig. 9B). This downregulation of Nrf2 was further corroborated by immunohistochemical analysis (Fig. 9C). Consistent with the in vivo findings, siRNA-mediated knockdown of SFTA2 in vitro also led to a corresponding decrease in SFTA2 and Nrf2 protein expressions (Fig. 9D–E), confirming a robust regulatory link between SFTA2 and the Nrf2 pathway.

### SFTA2 up-regulation reduced Nrf2 ubiquitination in the model of CRC

**Fig. 10:**
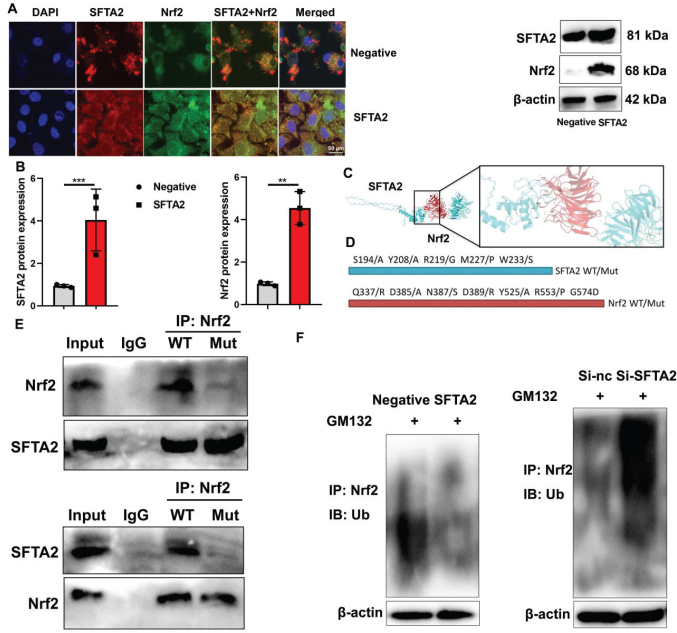
SFTA2 up-regulation reduced Nrf2 ubiquitination in the model of colorectal cancer. SFTA2/ Nrf2 protein expression (immunofluorescence, A), SFTA2/ Nrf2 protein expression (B) in vitro model by SFTA2 up-regulation; Hdock was used to dock SFTA2 and Nrf2 protein (C), SFTA2 / Nrf2 WT/Mut (D), IP assay for SFTA2 protein interlinking with Nrf2 protein (D), Nrf2 ubiquitination (E). Data were expressed as mean ± SD; the number of vitro model = 3.

We next elucidated the mechanism by which SFTA2 regulates Nrf2 activity in mitochondrial oxidation-induced ferroptosis. In CRC cells, SFTA2 overexpression induced SFTA2 and Nrf2 expression (Fig. 10A–B). A computational 3D model predicted a direct protein–protein interaction between SFTA2 and Nrf2 (Fig. 10C–D). IP analysis demonstrated that the SFTA2 WT protein interacts with the SFTA2 WT protein, while the SFTA2 WT protein does not interact with the Nrf2 Mut protein, and the Nrf2 Mut protein does not link with the SFTA2 WT protein (Fig. 10E). Furthermore, SFTA2 up-regulation reduced Nrf2 ubiquitination in the CRC model (Fig. 10F), indicating that SFTA2 enhances Nrf2 stability by inhibiting its proteasomal degradation.

### Nrf2 reversed the effects of si-SFTA2 on ferroptosis of CRC

**Fig. 11:**
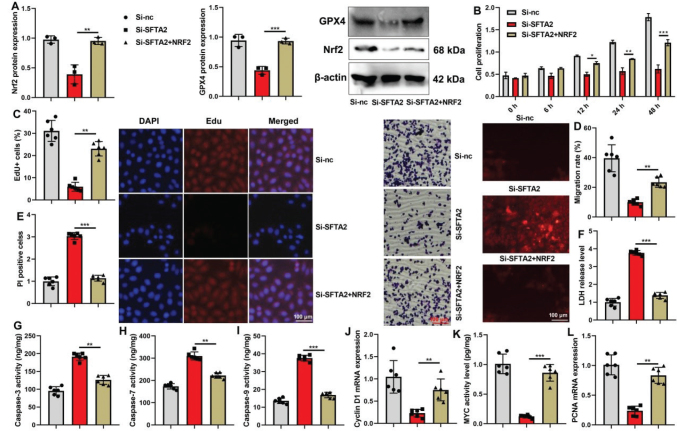
Nrf2 reversed the effects of si-SFTA2 on the cell proliferation of colorectal cancer. Nrf2/ GPX4 protein expression (A), cell growth (B), Migration (C), Edu positivity (D), PI levels (E), LDH activity (F), caspase-3/7/9 activity levels (G, H, I), Cyclin D1/ myc /PCNA mRNA expression (J, K, L) in colorectal cancer cells. **P < 0.01, ***P < 0.001. Data were expressed as mean ± SD; the number of mice model = 6; the number of vitro model = 3 or 6.

**Fig. 12:**
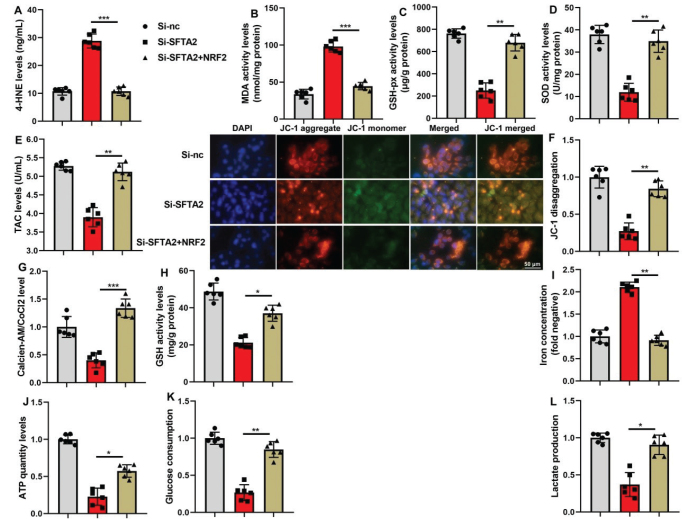
Nrf2 reversed the effects of si-SFTA2 on ferroptosis of colorectal cancer. 4-HNE, MDA, SOD, GSH-PX, and TAC activity levels (A, B, C, D, E), JC-1 assay (F), mitochondria CoCl2 levels (G), GSH activity level (H), GPX4 protein expression (I), ATP quantity levels (J), glucose consumption (K), lactate production (L) in vitro model by SFTA2 up-regulation. *P < 0.05, **P < 0.01, ***P < 0.001. Data were expressed as mean ± SD; the number of vitro model = 6.

Furthermore, we conducted rescue experiments to confirm that SFTA2 regulates ferroptosis primarily through the Nrf2 pathway. The application of an Nrf2 agonist effectively reversed the suppressive effects of si-SFTA2 on the protein expression of the Nrf2/GPX4 axis and restored cell proliferation in CRC cells (Fig. 11). Similarly, the agonist mitigated the impact of si-SFTA2 on mitochondrial dysfunction and the TCA cycle (Fig. 12), demonstrating that Nrf2 activation is sufficient to counteract the ferroptotic and metabolic consequences of SFTA2 knockdown.

## Discussion

The prognosis of colon cancer is critically influenced by postoperative recurrence and metastasis, underscoring the clinical importance of identifying reliable prognostic markers (29). As a malignant lesion originating from the colonic mucosal epithelium, colon cancer predominantly affects individuals over 40 years of age and occurs more frequently in men than in women (30). Its onset is often insidious, with nonspecific early symptoms; however, as the disease advances, patients may experience abdominal pain, weight loss, altered bowel habits, and changes in stool characteristics (31). However, as the disease progresses, symptoms such as emaciation, abdominal pain, weight loss, changes in defecation habits, and abnormal fecal properties may appear. Surgical resection remains the cornerstone of treatment for stages I–III colon cancer and has contributed to an overall 5-year survival rate exceeding 60% (32). Despite a high surgical success rate and significant improvements in postoperative survival and quality of life, over 30% of patients undergoing curative-intent surgery eventually develop recurrence or metastasis. Among these patients, the 5-year survival rate drops markedly to only 19% (29). Therefore, consistent postoperative surveillance is essential for improving outcomes and reducing mortality (33). In this study, we observed that SFTA2 was upregulated at both the mRNA and protein levels in CRC samples and cell lines, and was specifically expressed in cancer cells. Jennifer Luyapan et al. showed that SFTA2 influences lung cancer risk (34), suggesting it may function as a cancer-related gene across multiple tumor types. SFTA2 may be a potential target for diagnosis or treatment in the future. This study only collected 24 cases, which is a limitation of this study. In subsequent research, we will have a large sample size.

The oncogenic effects of c-Met are mediated through multiple mechanisms, including the regulation of cell proliferation, apoptosis, and the promotion of tumor cell invasion. Notably, c-Met can be activated through HGF-independent pathways, particularly via overexpression (35). Such overexpression may result from gene amplification, transcriptional upregulation, or post-transcriptional mechanisms, while impaired protein processing or loss of negative regulators can also lead to constitutive Met activation and tumorigenesis (36). Structurally, the mature Met protein consists of α and β subunits generated by proteolytic cleavage of a single-chain precursor (37). However, in the colon cancer LOVO cell line, defective post-translational processing results in the persistent presence of a single-chain precursor form on the cell surface, which exhibits sustained tyrosine kinase activity (38). Extensive studies have confirmed that HGF/c-Met co-expression occurs in numerous malignancies, where it promotes tumor proliferation, differentiation, and metastasis (39). In the tumor microenvironment, cancer cells can stimulate fibroblasts to secrete HGF via various cytokines (40), which then binds c-Met, induces receptor dimerization, and activates intracellular tyrosine kinase activity. This signaling not only disrupts intercellular junctions but also facilitates tumor infiltration and metastasis, underscoring the pivotal role of HGF/c-Met in tumor progression. In line with this, we observed that SFTA2 down-regulation reduced tumor volume and weight and decreased Myc mRNA expression in tumor tissues. Sevcan Atay et al. reported that SFTA2 might be correlated with c-Met in pancreatic ductal adenocarcinoma (41). Therefore, we propose that SFTA2 promotes CRC growth, potentially by upregulating c-Met expression. Ferroptosis is a form of regulated cell death initiated by cellular redox imbalance and heightened metabolic activity, culminating in iron-dependent lipid peroxidation and eventual cell demise (42). Distinct from other programmed death pathways such as apoptosis, ferroptosis is defined by its unique iron-dependent mechanism and pervasive lipid peroxidation (43). Growing evidence indicates that ferroptosis is intimately involved in tumorigenesis and cancer progression, making its targeted activation a promising therapeutic strategy for inducing tumor cell death (44, 45). In this context, our findings demonstrate that SFTA2 up-regulation suppresses ferroptosis in CRC by attenuating mitochondrial damage and restoring TCA cycle function.

Ferroptosis is a recently identified form of programmed cell death driven by intracellular Fe²^+^ overload, accumulation of ROS, and extensive lipid peroxidation (46). In colon cancer, the Nrf2/GPX4 signaling axis serves as a critical regulator of this process (47). Under physiological conditions, Nrf2 binds to Keap1 and remains in an inactive state; when cells are stimulated by hypoxia, Nrf2 is released from Keap1 and translocates into the nucleus, activating the expression of antioxidant enzymes such as HO-1, SLC7A11, and GPX4, thereby enhancing the antioxidant capacity of cells. Existing studies have shown that activating the Nrf2 pathway can alleviate ferroptosis in colon cancer cells (48). In line with this, our study revealed that SFTA2 up-regulation reduces Nrf2 ubiquitination in CRC models, suggesting a novel mechanism by which SFTA2 stabilizes Nrf2 to suppress ferroptosis. This study did not investigate the molecular mechanism by which SFTA2 promotes Nrf2 ubiquitination (such as the involvement of Keap1, Cul3, or other E3 ligases). This is a limitation of the present study. In future research, more experimental approaches should be used, such as co-immunoprecipitation profiling and domain mutation, to explore the specific domain of SFTA2 that regulates Nrf2.

**Fig. 13:**
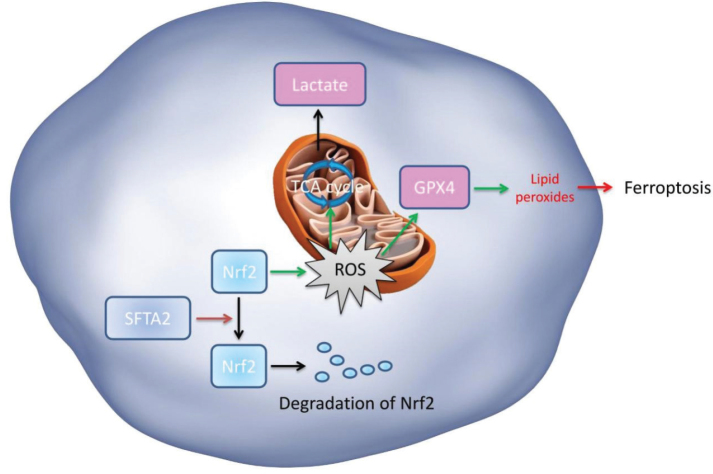
SFTA2 reduced colorectal cancer ferroptosis promoting metastasis through regulating EMT transition by degradation of Nrf2.

In conclusion, our findings demonstrate that SFTA2 activates the Nrf2 signaling pathway by inhibiting its ubiquitination, thereby attenuating mitochondrial damage and TCA cycle dysfunction in CRC (Fig. 13). Further elucidating the role of SFTA2 in regulating mitochondrial damage-induced ferroptosis for CRC, SFTA2 may act as an oncogene for CRC. Targeting SFTA2 may thus represent a promising strategy for the treatment of CRC and potentially other malignancies.

## Declarations

### Clinical trial

Not applicable.

## Ethics approval and consent to participate

All animal experiments were approved by the Ethical Committee of Jiujiang First People’s Hospital, and strictly implemented in compliance with the NIH Guide for the Care and Use of Laboratory Animals. All procedures were performed in accordance with ARRIVE guidelines.

The study was conducted in accordance with the Declaration of Helsinki and its subsequent amendments. The study was approved by the ethics committee of Jiujiang First People’s Hospital and written informed consent was taken from all the patients.

## Competing interests

The authors declare that they have no conflict of interest.

## Availability of data and material

The data sets used and analyzed in the current study are available on reasonable request from the corresponding authors.

## Authors’ contributions

JH and GHW developed the study concept and revised the manuscript accordingly. JH and GHW analyzed and interpreted the data. JH and SXM conducted the experiments and data analysis, and were involved in the preparation of the figures and manuscript. GHW and SXM drafted the manuscript. All authors contributed to the editing of the manuscript and approved the submitted version.

## References

[1] Jiao P, Wang S, Wu J, Zhao Y, Han J, Sha Z. Tislelizumab-induced hemophagocytic lymphohistiocytosis in a patient with microsatellite instability-high colon cancer and coexisting systemic lupus erythematosus: a case report and literature review. Front Oncol 2025; 15: 1585133. doi: 10.3389/fonc.2025.1585133 40842582 PMC12364699

[2] Kim S, Clark JI. Immunotherapy with pembrolizumab for resectable dMMR/MSI-H stage III colon cancer: a case of personalized, precision surgery-sparing immunotherapy. J Investig Med High Impact Case Rep 2025; 13: 23247096251368093. doi: 10.1177/23247096251368093 PMC12374102 40842405

[3] Liu T, Zhong L, Sun X, He Z, Lv W, Deng L, et al. Machine learning-driven multi-targeted drug discovery in colon cancer using biomarker signatures. NPJ Precis Oncol 2025; 9: 297. doi: 10.1038/s41698-025-01058-6 40847045 PMC12373948

[4] Pu Z, Zhang W, Wang M, Xu M, Xie H, Zhao J. Schisandrin B attenuates colitis-associated colorectal cancer through SIRT1 linked SMURF2 signaling. Am J Chin Med 2021; 49: 1773–89. doi: 10.1142/s0192415x21500841 34632965

[5] Okechukwu CC, Gmeiner WH. CF10/LV overcomes acquired resistance to 5-FU/LV in colorectal cancer cells through downregulation of the c-Myc/ABCB5 axis. Cancer Drug Resist 2025; 8: 35. doi: 10.20517/cdr.2025.76 40843355 PMC12367397

[6] Ouyang P, Gong J, Nie J, Kandala S, Shi Y, Tian Y, et al. Tamarixetin suppresses colorectal cancer progression by targeting DPP7-mediated WNT3A/β-Catenin signalling pathway. J Cell Mol Med 2025; 29: e70787. doi: 10.1111/jcmm.70787 40845164 PMC12372979

[7] Di Agostino S, La Padula D, Rago V, Gabriele C, Conforti F, Aprigliano E, et al. Proteomic profiling identifies a stromal TGF-β1/podoplanin axis as a driver of colorectal cancer progression. J Exp Clin Cancer Res 2025; 44: 247. doi: 10.1186/s13046-025-03496-3 40842027 PMC12372361

[8] Craig SG, Mende S, Humphries MP, Bingham V, Viratham Pulsawatdi A, Loughrey MB, et al. Orthogonal MET analysis in a population-representative stage II–III colon cancer cohort: prognostic and potential therapeutic implications. Mol Oncol 2021; 15: 3317–28. doi: 10.1002/1878-0261.13089 34428346 PMC8637556

[9] Gu Y, Chen Y, Wei L, Wu S, Shen K, Liu C, et al. ABHD5 inhibits YAP-induced c-Met overexpression and colon cancer cell stemness via suppressing YAP methylation. Nat Commun 2021; 12: 6711. doi: 10.1038/s41467-021-26967-5 34795238 PMC8602706

[10] Arlt F, Stein U. Colon cancer metastasis: MACC1 and met as metastatic pacemakers. Int J Biochem Cell Biol 2009; 41: 2356–9. doi: 10.1016/j.biocel.2009.08.001 19666136

[11] Hou S, Wang L, Yang C, Li Y, Liu H. MACC1 drives metastasis in colorectal cancer by coordinating YKT6-dependent exosome biogenesis and c-Met cargo selection. Cell Signal 2025; 135: 112073. doi: 10.1016/j.cellsig.2025.112073 40834974

[12] El-Desouky SI, Nafie MS, Haffez H, Moustafa MAA, Ali AR. Scaffold-hopping strategy for pyrazolo[3,4-d]pyrimidines: in vitro and in silico studies of dual c-Met/STAT3 inhibition for enhanced antitumor activity. Bioorg Chem 2025; 164: 108821. doi: 10.1016/j.bioorg.2025.108821 40782411

[13] Recondo G, Che J, Jänne PA, Awad MM. Targeting MET dysregulation in cancer. Cancer Discov 2020; 10: 922–34. doi: 10.1158/2159-8290.cd-19-1446 32532746 PMC7781009

[14] Kim HJ. Therapeutic strategies for ovarian cancer in point of HGF/c-MET targeting. Medicina (Kaunas) 2022; 58: 649. doi: 10.3390/medicina58050649 35630066 PMC9147666

[15] Guo R, Luo J, Chang J, Rekhtman N, Arcila M, Drilon A. MET-dependent solid tumours – molecular diagnosis and targeted therapy. Nat Rev Clin Oncol 2020; 17: 569–87. doi: 10.1038/s41571-020-0377-z 32514147 PMC7478851

[16] Yang X, Liao HY, Zhang HH. Roles of MET in human cancer. Clin Chim Acta 2022; 525: 69–83. doi: 10.1016/j.cca.2021.12.017 34951962

[17] Fu J, Su X, Li Z, Deng L, Liu X, Feng X, et al. HGF/c-MET pathway in cancer: from molecular characterization to clinical evidence. Oncogene 2021; 40: 4625–51. doi: 10.1038/s41388-021-01863-w 34145400

[18] Zheng X, Wang Q, Zhou Y, Zhang D, Geng Y, Hu W, et al. N-acetyltransferase 10 promotes colon cancer progression by inhibiting ferroptosis through N4-acetylation and stabilization of ferroptosis suppressor protein 1 (FSP1) mRNA. Cancer Commun (London) 2022; 42: 1347–66. doi: 10.1002/cac2.12363 PMC9759759 36209353

[19] Pu Z, Gui Y, Wang W, Shui Y, Xie H, Zhao M. Ophiopogonin D from ophiopogon japonicas-induced USP25 activity to reduce ferroptosis of macrophage in acute lung injury by the inhibition of bound Rac1 and Nox1 complex. Am J Chin Med 2025; 53: 501–22. doi: 10.1142/s0192415x25500193 40099394

[20] Dixon SJ, Lemberg KM, Lamprecht MR, Skouta R, Zaitsev EM, Gleason CE, et al. Ferroptosis: an iron-dependent form of nonapoptotic cell death. Cell 2012; 149: 1060–72. doi: 10.1016/j.cell.2012.03.042 22632970 PMC3367386

[21] Wu Z, Lu Z, Li L, Ma M, Long F, Wu R, et al. Identification and validation of ferroptosis-related LncRNA signatures as a novel prognostic model for colon cancer. Front Immunol 2021; 12: 783362. doi: 10.3389/fimmu.2021.783362 35154072 PMC8826443

[22] Stockwell BR, Friedmann Angeli JP, Bayir H, Bush AI, Conrad M, Dixon SJ, et al. Ferroptosis: a regulated cell death nexus linking metabolism, redox biology, and disease. Cell 2017; 171: 273–85. doi: 10.1016/j.cell.2017.09.021 28985560 PMC5685180

[23] Pu Z, Li L, Zhang Y, Shui Y, Liu J, Wang X, et al. Exploring the therapeutic potential of HAPC in COVID-19-induced acute lung injury. Phytomedicine 2025; 139: 156563. doi: 10.1016/j.phymed.2025.156563 40023068

[24] Zheng Y, Li L, Chen H, Zheng Y, Tan X, Zhang G, et al. Luteolin exhibits synergistic therapeutic efficacy with erastin to induce ferroptosis in colon cancer cells through the HIC1-mediated inhibition of GPX4 expression. Free Radic Biol Med 2023; 208: 530–44. doi: 10.1016/j.freeradbiomed.2023.09.014 37717793

[25] Pu Z, Gui Y, Wang W, Shui Y, Xie H, Zhao M. Ophiopogonin D from Ophiopogon japonicas-induced USP25 activity to reduce ferroptosis of macrophage in acute lung injury by the inhibition of bound Rac1 and Nox1 complex. Am J Chin Med 2025; 53: 501–22. doi: 10.1142/S0192415X25500193.2025.03.19 40099394

[26] Pu Z, Wang W, Xie H, Wang W. Apolipoprotein C3 (ApoC3) facilitates NLRP3 mediated pyroptosis of macrophages through mitochondrial damage by accelerating of the interaction between SCIMP and SYK pathway in acute lung injury. Int Immunopharmacol 2024; 128: 111537. doi: 10.1016/j.intimp.2024.111537 38232538

[27] Pu Z, Sui B, Wang X, Wang W, Li L, Xie H. The effects and mechanisms of the anti-COVID-19 traditional Chinese medicine, Dehydroandrographolide from Andrographis paniculata (Burm.f.) Wall, on acute lung injury by the inhibition of NLRP3-mediated pyroptosis. Phytomedicine 2023; 114: 154753. doi: 10.1016/j.phymed.2023.154753 37084628 PMC10060206

[28] Pu Z, Shen C, Zhang W, Xie H, Wang W. Avenanthramide C from oats protects pyroptosis through dependent ROS-induced mitochondrial damage by PI3K ubiquitination and phosphorylation in pediatric pneumonia. J Agric Food Chem 2022; 70: 2339–53. doi: 10.1021/acs.jafc.1c06223 35119859

[29] Wang Y, Zhang J, Mou C, Hu Y, He Z, Lee J, et al. Protopanaxatriol, a ginsenoside metabolite, induces apoptosis in colorectal cancer cells and arrests their cell cycle by targeting AKT. J Ginseng Res 2025; 49: 488–501. doi: 10.1016/j.jgr.2025.03.012 40843020 PMC12365516

[30] Wang X, Pi L, Chen Y, Tan J, Wang Y, Xia K, et al. Exploring the multifaceted roles of glutamate oxaloacetate transaminase 1 as a biomarker and therapeutic target in colorectal cancer and pan-cancer analyses. Discov Oncol 2025; 16: 1600. doi: 10.1007/s12672-025-03461-8 40849558 PMC12374927

[31] Yuan X, Wang Q, Zhao J, Xie H, Pu Z. The m6A methyltransferase METTL3 modifies Kcnk6 promoting on inflammation associated carcinogenesis is essential for colon homeostasis and defense system through histone lactylation dependent YTHDF2 binding. Int Rev Immunol 2025; 44: 1–16. doi: 10.1080/08830185.2024.2401358 39269733

[32] Zhang W, Gu Y, Xue X, Li X, Wang W, Wang R, et al. Prognostic significance of DNA ploidy, stroma fraction, and nucleotyping in stage IIIb colon cancer and their implications for adjuvant chemotherapy. Clin Transl Oncol 2026; 28: 625–34. doi: 10.1007/s12094-025-04029-2 40841502

[33] Platt JR, Elliott F, Handley K, Magill L, Quirke P, Seymour MT, et al. CT staging performance in an international trial of neoadjuvant chemotherapy for locally advanced colon cancer. Br J Radiol 2025; 98: 2175–83. doi: 10.1093/bjr/tqaf217 40848246 PMC12810875

[34] Luyapan J, Bossé Y, Li Z, Xiao X, Rosenberger A, Hung RJ, et al. Candidate pathway analysis of surfactant proteins identifies CTSH and SFTA2 that influences lung cancer risk. Hum Mol Genet 2023; 32: 2842–55. doi: 10.1093/hmg/ddad095 37471639 PMC10481107

[35] Migliore C, Martin V, Leoni VP, Restivo A, Atzori L, Petrelli A, et al. MiR-1 downregulation cooperates with MACC1 in promoting MET overexpression in human colon cancer. Clin Cancer Res 2012; 18: 737–47. doi: 10.1158/1078-0432.ccr-11-1699 22179665

[36] Song N, Liu S, Zhang J, Liu J, Xu L, Liu Y, et al. Cetuximab-induced MET activation acts as a novel resistance mechanism in colon cancer cells. Int J Mol Sci 2014; 15: 5838–51. doi: 10.3390/ijms15045838 24714091 PMC4013599

[37] Luraghi P, Reato G, Cipriano E, Sassi F, Orzan F, Bigatto V, et al. MET signaling in colon cancer stem-like cells blunts the therapeutic response to EGFR inhibitors. Cancer Res 2014; 74: 1857–69. doi: 10.1158/0008-5472.can-13-2340-t 24448239

[38] Mezquita B, Pineda E, Mezquita J, Mezquita P, Pau M, Codony-Servat J, et al. LoVo colon cancer cells resistant to oxaliplatin overexpress c-MET and VEGFR-1 and respond to VEGF with dephosphorylation of c-MET. Mol Carcinog 2016; 55: 411–19. doi: 10.1002/mc.22289 25647613

[39] Tsai HL, Tsai YC, Chen YC, Huang CW, Chen PJ, Li CC, et al. MicroRNA-148a induces apoptosis and prevents angiogenesis with bevacizumab in colon cancer through direct inhibition of ROCK1/c-Met via HIF-1α under hypoxia. Aging 2022; 14: 6668–88. doi: 10.18632/aging.204243 35997665 PMC9467409

[40] Liu QQ, Zeng XL, Guan YL, Lu JX, Tu K, Liu FY. Verticillin A inhibits colon cancer cell migration and invasion by targeting c-Met. J Zhejiang Univ Sci B 2020; 21: 779–95. doi: 10.1631/jzus.B2000190 33043644 PMC7606203

[41] Atay S. Integrated transcriptome meta-analysis of pancreatic ductal adenocarcinoma and matched adjacent pancreatic tissues. PeerJ 2020; 8: e10141. doi: 10.7717/peerj.10141 33194391 PMC7597628

[42] Wang B, Kong W, Lv L, Wang Z. Plumbagin induces ferroptosis in colon cancer cells by regulating p53-related SLC7A11 expression. Heliyon 2024; 10: e28364. doi: 10.1016/j.heliyon.2024.e28364 38596137 PMC11002553

[43] Liu J, Wei X, Xie Y, Yan Y, Xue S, Wang X, et al. MDM4 inhibits ferroptosis in p53 mutant colon cancer via regulating TRIM21/GPX4 expression. Cell Death Dis 2024; 15: 825. doi: 10.1038/s41419-024-07227-y 39543140 PMC11564821

[44] Wu J, Liu C, Wang T, Liu H, Wei B. Deubiquitinase inhibitor PR-619 potentiates colon cancer immunotherapy by inducing ferroptosis. Immunology 2023; 170: 439–51. doi: 10.1111/imm.13683 37526037

[45] Li Y, Liu J, Chen Y, Weichselbaum RR, Lin W. Nanoparticles synergize ferroptosis and cuproptosis to potentiate cancer immunotherapy. Adv Sci (Weinh) 2024; 11: e2310309. doi: 10.1002/advs.202310309 38477411 PMC11187894

[46] Li Y, Tuerxun H, Liu X, Zhao Y, Wen S, Li Y, et al. Nrf2 – a hidden bridge linking cancer stem cells to ferroptosis. Crit Rev Oncol Hematol 2023; 190: 104105. doi: 10.1016/j.critrevonc.2023.104105 37598896

[47] Luo X, Wang Y, Zhu X, Chen Y, Xu B, Bai X, et al. MCL attenuates atherosclerosis by suppressing macrophage ferroptosis via targeting KEAP1/NRF2 interaction. Redox Biol 2024; 69: 102987. doi: 10.1016/j.redox.2023.102987 38100883 PMC10761782

[48] Wei R, Zhao Y, Wang J, Yang X, Li S, Wang Y, et al. Tagitinin C induces ferroptosis through PERK-Nrf2-HO-1 signaling pathway in colorectal cancer cells. Int J Biol Sci 2021; 17: 2703–17. doi: 10.7150/ijbs.59404 34345202 PMC8326123

